# Pan-vaccinomics strategy for developing a universal multi-epitope vaccine against endocarditis-related pathogens

**DOI:** 10.3389/fimmu.2025.1524128

**Published:** 2025-04-11

**Authors:** Peng Chao, Xueqin Zhang, Lei Zhang, Wei Ma, Dong Wang, Aiping Yang, Xiaoyang Chen

**Affiliations:** ^1^ Department of Cardiovascular Disease, People’s Hospital of Xinjiang Uygur Autonomous Region, Urumqi, China; ^2^ Department of Nephropathy Disease, People’s Hospital of Xinjiang Uygur Autonomous Region, Urumqi, China; ^3^ Department of Endocrine Disease, People’s Hospital of Xinjiang Uygur Autonomous Region, Urumqi, China; ^4^ Department of Electrocardiogram, People’s Hospital of Xinjiang Uygur Autonomous Region, Urumqi, China

**Keywords:** to Combat global antibiotic resistance endocarditis, pathogenic bacteria, core proteome, multi-epitope vaccine, cholera toxin

## Abstract

**Introduction:**

Endocarditis is a life-threatening infection of the heart valves, frequently caused by pathogenic bacteria. The growth of multidrug-resistant bacteria necessitates the development of innovative therapeutic techniques, such as vaccines.

**Methods:**

The current study employed core proteome analysis and a computational-based reverse vaccinology approach across multiple bacterial pathogens associated with endocarditis to identify prospective universal vaccine candidates. The core proteome analysis contained 121 highly pathogenic strains from ten distinct pathogens (*Streptococcus mutans*, *Streptococcus viridans*, *Streptococcus pyogenes*, *Staphylococcus aureus*, *Enterococcus faecalis*, *Streptococcus agalactiae*, *Gemella morbillorum*, *Streptococcus pneumonia*, *Enterococcus faecium*, and *Streptococcus gallolyticus*). The core proteome was subjected to a subtractive proteomics methodology.

**Results:**

Three proteins that were virulent, non-homologous, antigenic, and non-allergenic have been identified as prospective candidates for vaccine development: 30S ribosomal protein S13, 50S ribosomal protein L6, and UMP Kinase. B and T cell epitopes were predicted from vaccine candidate proteins using a range of immune-informatics methods. An in silico vaccine was created by using meticulously chosen epitopes—seven Cytotoxic T lymphocyte (CTL), seven Linear B lymphocyte (LBL), and three Helper T lymphocyte (HTL) epitopes—and subsequently aligning them with the major histocompatibility complex (MHC) molecules (MHC I & MHC II) and human TLR4. A Cholera toxin subunit B (CTB) adjuvant was added to the vaccine to enhance the immunological response. The molecular interactions and binding affinity of the vaccine with TLR4 and MHC molecules were analyzed using molecular dynamics (MD) simulations and molecular docking. To ensure optimal vaccine protein expression, the vaccine was cloned and reverse-translated in *E. coli*.

**Discussion:**

This methodology tackles the difficulties presented by the diversity of pathogens and antibiotic resistance, providing a strategic option for developing efficient and durable vaccines against infections associated with endocarditis.

## Graphical Abstract

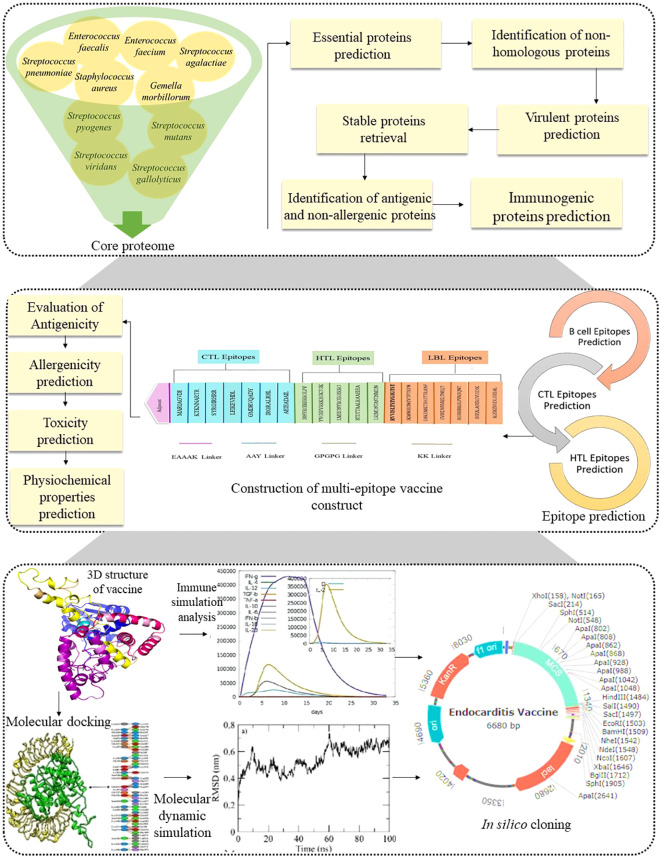

## 1 Introduction

Endocarditis is a severe medical condition characterized by the inflammation of the endocardium (interior lining of the heart chambers and valves). The primary cause of this illness is the introduction of bacteria, fungi, or other microbes into the bloodstream, which then adhere to the heart. Endocarditis, while uncommon, can result in serious consequences and has the potential to be life-threatening if not rapidly and adequately treated (1, 2). It is estimated that the annual incidence of endocarditis is 3 to 10 cases per 100,000 individuals. This disease process has traditionally exhibited a higher prevalence in men compared to women, with a ratio of approximately 2:1. Most people who get bacterial endocarditis are now older than 65 years old (3). This higher rate in older people is probably because more people in that age group have risk factors like indwelling cardiac devices, artificial valves, hemodialysis, acquired valvular disease, and diabetes mellitus (4). Before antibiotics came along, rheumatic heart disease was a big risk factor, but now it only accounts for less than 5% of all cases. Approximately 10% of all occurrences of infectious endocarditis are now associated with recreational intravenous drug use, making it a growing risk factor (5, 6).

Endocarditis symptoms can range from mild to severe, appearing gradually over weeks or suddenly over days. Typical signs and symptoms encompass persistent fever, chills, fatigue, musculoskeletal pain, nocturnal sweating, dyspnea, and edema in the feet, legs, or abdomen. Patients may also suffer new or modified heart murmurs, which are abnormal heart sounds detected using a stethoscope (2, 7).

Endocarditis epidemiology differs by continent and regions because of variations in risk factors, pathogen prevalence, and access to healthcare. Between 3 and 10 instances per 100,000 people are reported each year in North America and Europe, with *Staphylococcus aureus* being the most common cause, particularly in cases related to healthcare. A greater burden of streptococcal endocarditis is caused by rheumatic heart disease (RHD), which is still a significant risk factor in Asia and Africa. While endocarditis is more common in Oceania among indigenous cultures because of a higher prevalence of RHD, it is frequently associated with Chagas disease in Latin America. The increasing prevalence of intravenous drug use, immunosuppressive disorders, and infections of prosthetic valves is impacting the changing epidemiology of endocarditis in all regions (8–10).

A bacterial infection is the primary cause of endocarditis. Bacteria can get access to the bloodstream by multiple pathways, such as infections in other parts of the body, dental treatments, specific medical interventions, and intravenous drug administration. Once introduced into the bloodstream, these bacteria can migrate to the heart and attach themselves to injured sites or faulty cardiac valves. This results in the formation of vegetation, which is composed of blood clots, bacteria, and cellular debris (11, 12). Gram-positive streptococci, enterococci, and staphylococci represent the primary etiological agents of infected endocarditis. ‘The three pathogens together cause 80–90% of infections, with *Staphylococcus aureus* being responsible for approximately 30% of cases in developed regions (6).

Endocarditis treatment primarily entails administering antibiotics and, in more severe instances, surgical intervention. Intravenous antibiotics are given for several weeks in order to treat the disease. The type of bacteria causing the infection determines the treatment option and length of time. Blood cultures are crucial for identifying the infection so that a targeted antibiotic treatment can be administered. If the infection has resulted in substantial damage to the heart valves, surgical intervention may be required to mend or substitute the afflicted valves. This is particularly accurate if the disease shows resistance to antibiotics, if there is a chronic infection despite therapy, or if the patient is in danger of heart failure due to valve malfunction (13–15).

Endocarditis prevention is especially important. Maintaining good oral hygiene and scheduling routine dental treatment are crucial preventive steps since mouth germs can enter the bloodstream and impact the heart. Prophylactic antibiotics may occasionally be recommended before certain dental or surgical procedures to prevent infection. Avoiding intravenous drug use and following safe protocols when receiving medical treatments can help reduce the risk of endocarditis (15, 16).

Despite these precautions, endocarditis remains a difficult condition to manage due to the risk of serious consequences and the growing incidence of antibiotic-resistant bacteria. This is where the necessity for a vaccine is vital. A vaccination could provide a preventive defense against the microorganisms that cause endocarditis, preventing bacteria from entering the bloodstream and causing disease (17). Given the variety of microorganisms that can cause endocarditis, a multi-epitope vaccine targeting many important pathogens could be especially beneficial. Consequently, a multi-epitope vaccine was developed in this study targeting pathogens responsible for endocarditis through an immunoinformatics methodology and *in silico* design.

## 2 Materials and methods

### 2.1 Retrieval of proteomic data

Initially, proteomic data for 121 strains of ten pathogens (*Streptococcus mutans*, *Streptococcus viridans*, *Streptococcus pyogenes*, *Staphylococcus aureus*, *Enterococcus faecalis*, *Streptococcus agalactiae, Gemella morbillorum*, *Streptococcus pneumonia*, *Enterococcus faecium*, and *Streptococcus gallolyticus*) that causes endocarditis were obtained from Genbank (18). The 121 highly pathogenic strains evaluated in the core proteome analysis are prevalent around the world and have been associated to infective endocarditis (IE) in various locales. *Streptococcus mutans* and *Streptococcus viridans*, members of the oral microbiota, have a role in community-acquired endocarditis worldwide, particularly in North America, Europe, and Asia (19, 20). In affluent countries, *Streptococcus pyogenes* and *Streptococcus agalactiae* are more common, and invasive soft tissue infections can cause secondary endocarditis (21, 22). *Staphylococcus aureus* is a major cause of endocarditis associated with healthcare and intravenous drug use, and it is widely distributed in North America, Europe, and Australia (23). Antibiotic resistance is becoming a major concern in Asia and Africa. *Enterococcus faecalis* and *Enterococcus faecium* are widespread, with high incidence of hospital-acquired infections in the United States, Europe, and Latin America (24, 25). *Streptococcus pneumoniae*, primarily a respiratory pathogen, has been linked to endocarditis cases in Africa and Asia, while *Streptococcus gallolyticus*, which is frequently linked to colorectal cancer and endocarditis, is more commonly reported in Western countries (26, 27). *Gemella morbillorum* is less common but has been documented in North America and Europe (28). The effect of endocarditis is highlighted by its diverse geographic distribution, which also highlights the need for regionally specific surveillance and preventative measures.

The proteomes underwent analysis through the Bacterial pan-genome analysis program (BPGA) to discern proteins that are universally shared among the pathogens and exhibit a degree of conservation (29). Core proteome was generated through BPGA, and this was done to prioritize prospective vaccine candidates.

### 2.2 Vaccine candidate prioritization and subtractive proteomics

Certain proteins may be necessary for cell survival. The essential proteins were identified by uploading the core proteome to the Geptop server and analyzing it with a threshold of 0.2 (30). Human homologs were eliminated by an examination of essential proteins, and non-essential proteins were eliminated as well. The human proteome was screened for essential proteins using NCBI’s BLASTp with a 10^-4^ expectation value (31). The removal of human homolog proteins was implemented to prevent the potential activation of an autoimmune response within the host organism. In the process of vaccine design, it is imperative to take into account the detrimental impacts of virulent proteins. The Virulent Factor Database (VFDB) was utilized to discern pathogenic proteins (32). The VFDB homologs of pathogenic proteins exhibited identities over 30% and bit scores surpassing 100. The Vaxijen server was also used to prove that pathogenic proteins were antigenic (33). Antigenic proteins with high scores were chosen to be studied further in order to make vaccines. The protein’s allergenicity was evaluated using the AllerTOP server (34). Utilizing the ProtParam tool, the pathogen’s secretome and exoproteome were selected and physiochemically analyzed to facilitate experimental investigations of the vaccine (35). Proteins with a molecular weight (M.W) below 110 kDa are more favorable candidates for potential vaccine targets. The TMHMM server was utilized to assess the number of transmembrane helices in the selected proteins (36). Proteins possessing either one or no transmembrane helix were selected for subsequent analysis. The InterProScan server was used to anticipate the biological processes and molecular activities of the target proteins (37).

### 2.3 Prediction & selection of epitopes

Epitope selection required B-cell and T-cell (HTL and CTL) prediction. To determine Cytotoxic T lymphocyte (CTL), Linear B lymphocyte (LBL), and Helper T lymphocyte (HTL) epitopes, the Immune Epitope Database (IEDB) was utilized, which includes MHC-I and MHC-II binding tools (38). The Immune Epitope Database service analyzed all selected proteins for MHC-I alleles, including 32 HLA-B, 18 HLA-A, and 20 HLA-C alleles. Regardless of MHC genotype, peptide epitopes might have varying length preferences. However, we focused on 9-mer epitopes since they are the best length for binding to MHC class I molecules, which play an important role in immune response activation. This study employed a consensus technique, 15-mer peptide length selection, and a reference set of 27 human leukocyte antigen (HLA) alleles to forecast HTL epitopes using the IEDB. To identify MHC-I and MHC-II epitopes with strong binding, we employed a threshold of adjusted rank < 2.

B-cell epitopes are important in vaccine development because they elicit a humoral immune response by attaching to B cell receptors. This activation causes B cells to generate antibodies that help destroy infections and give immunity. The ABCpred server predicted linear B-cell epitopes using antigen analysis (39). Epitope detection was conducted utilizing a recurrent neural network characterized by a 16-amino-acid window length and a threshold value set at 0.51.

The predicted epitopes (MHC-I and B-cell) were validated using the Vaxijen (v2.0), ToxinPred, Allergen FP1.0, and the MHC class I immunogenicity servers (33, 39–41). The HTL epitopes chosen from the IEDB server underwent analysis to assess their capacity to stimulate IFN-γ in comparison to other cytokines, utilizing the IFNepitope web server for this evaluation (42). The capacity for IL-4 induction was analyzed through the IL4Pred web server, while the evaluation of IL-10 induction capability was conducted using the IL10Pred web server (43, 44). The IFNepitope, IL4pred, and IL-10Pred servers utilize SVM (Support Vector Machine) based prediction.

### 2.4 Epitopes population coverage

The diverse ethnic and regional composition influences the distribution and manifestation of HLA alleles, subsequently impacting the effectiveness of vaccines. The assessment of population coverage was conducted through the application of the IEDB population coverage tool, which also incorporated the HLA-binding alleles pertinent to the respective MHC Class-I and MHC Class-II epitopes (45). This tool assesses the demographic representation of each epitope across diverse global regions through a meticulous examination of the distribution of HLA-binding alleles.

### 2.5 Vaccine construction

The vaccine design was developed by integrating the CTL, HTL, and B-cell epitopes with suitable adjuvants and linkers. An adjuvant is a component of the vaccine that significantly enhances the capacity to elicit an immune response and requires careful selection. The adjuvant utilized in the vaccine construct was Cholera enterotoxin subunit B, identified by the accession number P01556. The EAAAK linker was used to join the additives together because it makes the whole structure more stable. After that, the AAY linker, the KK linker, and the GPGPG linker were used to connect the Cytotoxic T lymphocyte (CTL), Linear B lymphocyte (LBL), and Helper T lymphocyte (HTL) epitopes.

### 2.6 Post-vaccine analysis

#### 2.6.1 Identification of physiochemical characteristics

Initially, the developed vaccine underwent scrutiny using NCBI-BLASTp to determine its resemblance to the human proteome. Furthermore, the vaccine’s antigenicity and immunogenicity were assessed using the VaxiJen v2.0 server and the IEDB immunogenicity tool, respectively (33, 46). The primary objective of utilizing AllerTOP to assess the allergenicity of the vaccine was to prevent any adverse reactions (34). Subsequently, the ProtParam server was employed to evaluate the physicochemical properties of the vaccine construct, including the Grand Average of Hydropathicity (GRAVY), Aliphatic Index (AI), Instability Index (II), Theoretical Isoelectric Point (theoretical PI), and Molecular Weight (MW), etc. (35). The solubility of the vaccine construct was further confirmed through the use of the SolPro server (47).

#### 2.6.2 Validation and prediction of vaccine structure (secondary and tertiary)

It is important to evaluate the secondary structure of the vaccine construct as it is a crucial marker of protein folding; hence, the SOPMA tool was used (48). Accurately predicting the tertiary structure of the vaccine is essential in order to verify its stability and assess its docking analysis with a Toll-like receptor (TLR4). The Robetta server was chosen through a CAMEO evaluation process to produce a three-dimensional structure for the vaccine sequence (49). Robetta uses the comparative modelling technique called RoseTTAFold to build the tertiary structure when a template structure can be discovered for a given amino acid sequence using BLAST, PSI-BLAST, 3D-Jury or FFAS03. Robetta employs the *de novo* Rosetta fragment insertion approach to generate the structure in the event that a suitable template is not available (50). The GalaxyRefine server accelerated the process of molecular structure refinement, thereby improving the integrity of the structure (51).

The comprehensive quality score of the predicted structure was evaluated using the ProSA-web (52). The ERRAT server was utilized to assess the interactions among non-bonded atoms (53). The Ramachandran plot served as a tool for evaluating the dihedral angles (phi and psi) that may be either prohibited or permissible (54).

#### 2.6.3 Conformational B-cell epitopes prediction

The conformational B-cell epitopes for the multi-epitope vaccine design were examined following the prediction of the tertiary structure. The prediction was generated using the ElliPro Server, a reliable tool designed for the identification of B-cell epitopes targeting a specific antigen (55).

### 2.7 Disulfide engineering

An essential element of the molecular composition of peptides and proteins involves the formation of disulphide bridges between cysteine residues. Such interactions may contribute to the stabilization of proteins by elevating the free energy associated with the denatured state and diminishing the conformational entropy. In this study, the disulphide bond was identified using the online Disulphide by Design 2.0 (DbD2) server (56). The server can produce three-dimensional vaccine structures to identify any residual pairs that are capable of forming disulphide linkages.

### 2.8 Immune simulation

C-ImmSim, an *in-silico* approach, presented the immune response profile of vaccine construct, allowing the characterization of both cellular and humoral responses of the mammalian immune system caused by the chimeric vaccine (57). The vaccine was administered over four weeks, with three separate instances, using the default settings for all simulations. The time intervals used were 1 hour, 82 hours, and 126 hours, which were chosen based on the concept of 8-hour cell division cycles in real life. The simulations were conducted with a random seed of 12,345. The vaccine injection lacked lipopolysaccharide (LPS), with the simulation volume established at 50µl and the number of steps fixed at 1000.

### 2.9 Molecular docking

The vaccine prompts a robust immune response by engaging with the host’s immune cells. The possible interaction of the vaccine with human TLR4 and MHC molecules was investigated by means of protein-protein docking. The vaccine was docked with MHCI (PDB ID: 1I1Y), TLR4 (PDB ID: 4G8A), and MHCII (PDB ID: 1KG0) by the HADDOCK v.2.2 server (58). HADDOCK, a sophisticated docking technique grounded in information analysis, serves to model biomolecular complexes. The residues participating in interactions were identified by employing the PDBsum online database (59), and PyMOL was employed to visualize the docked complex (60).

### 2.10 MD simulation

A molecular dynamics simulation lasting 100 ns was conducted to assess the stability of docked complexes. The solvation of the complex was conducted within a periodic box measuring 10 Å, which was filled with TIP3P water molecules (61). The system was designed to reach neutralization by including counter ions of Na+ and Cl-. Using the steepest descend approach of 5000 steps after neutralization, steric conflicts were eliminated, so minimizing the system. After minimization, the systems were equilibrated for 50,000 and 100,000 steps, respectively, at 310 K inside the NPT and NVT ensembles (62). The Parrinello-Rahman algorithms and the Berendson thermostat were employed to maintain the temperature and pressure at 310 K and 1 abm, respectively, during the simulation. The system was relaxed by adjusting the duration to τ P = 2.0 ps and τ T = 0.1 ps. Then, Verlet computed the non-bonded interactions (63) while the hydrogen atom bond lengths were kept at their ideal lengths by means of the LINCS technique (64). The particle mesh Ewald method was applied to estimate the electrostatic interactions beyond the short-range limit (65). Applications of periodic boundary conditions in the x, y, and z directions preceded a system production run. Every 10 picoseconds, the trajectory of the production run was recorded and analyzed utilizing the GROMACS commands and R BIO3D package (66). The simulation was run with the CHARMM36 forcefield and the Gromacs simulation tool (67).

### 2.11 *In silico* cloning

Optimization of codons is crucial for the efficient expression of a foreign gene within a host organism while also taking into account the host’s distinctive characteristics. Codon optimization was conducted utilizing the VectorBuilder platform. The altered sequence of codons was introduced into the PET28a (+) *E. coli* expression vector by positioning it between the HindIII and XhoI restriction sites utilizing GenSmart (68).

## 3 Results

### 3.1 Core proteome analysis

Core proteins are currently recognized as significant components in vaccine formulations, as they are found in all or most strains of the target pathogen. Their incorporation into vaccines provides immune protection against a wider array of pathogenic species. To develop a vaccine against endocarditis, 121 pathogenic strains of ten pathogens were evaluated (**Additional File 1**). Upon completion of the core proteome analysis, the total protein count was 12285.

### 3.2 Identification of Vaccine Candidates

In the initial step, essential proteins were retrieved from core proteome. After uploading the data to the Geptop server, 48 proteins were estimated to be essential. To identify essential proteins that are distinct from their human homologs, Blast P was implemented. Among the 48 essential proteins, there were 16 non-homologous proteins. After evaluating these proteins as virulence factors, it was determined that seven of these proteins are pathogenic. In addition, their antigenicity, allergenicity, immunogenicity, stability, transmembrane helices, and biological and molecular processes were evaluated. Three proteins with high immunogenicity, non-allergenicity, antigenicity, stability, and 0 transmembrane helices were selected as targets for the vaccine. Their information is presented in **Table 1**.

**Table 1:** Details of three shortlisted vaccine candidates.

Sr.No	Name and Accession No	Antigenicity	Immunogenicity	Allergenicity	Transmembrane helices	Biological Processes	Molecular Processes	Cellular Processes
P1	30S ribosomal protein S13 (HCY1222769.1)	0.7	0.80	Non-Allergen	**0**	Translation	RNA binding structural constituent of ribosome nucleic acid binding	Ribosome
P2	50S ribosomal Protein L6 (WP_002356217.1)	0.7	1.15	Non-Allergen	**0**	Translation	RNA binding structural constituent of ribosome	Ribosome
P3	UMP Kinase (WP_006595852.1)	0.5	2.30	Non-Allergen	**0**	pyrimidine nucleotide biosynthetic process	UMP/dUMP kinase activity UMP kinase activity	Cytoplasm

### 3.3 Evaluation of epitopes

The methodology for vaccine design involved selecting seven cytotoxic T lymphocyte (CTL) epitopes from **Table 2**, five helper T lymphocyte (HTL) epitopes from **Table 3A**, and seven B lymphocyte (LBL) epitopes from **Table 3B**. The selection of these epitopes (B-cell and CTL) was based on immunogenicity, non-toxicity, antigenicity, and non-allergenicity. HTL epitopes was selected on the basis of their Ifn, IL4, and IL10 inducing ability.

**Table 2:** Top seven CTL epitopes selected for endocarditis vaccine.

Protein	Epitopes	Alleles	Position	Antigenicity	Immunogenicity	Allergenicity
P1	MARIAGVDI	HLA-B*51:01, HLA-A*30:01	1-9	0.7	0.26	Non-Allergenicity
	KTKNNARTR	HLA-A*30:01, HLA- A*31:01	101-109	2.1	0.02	Non-Allergenicity
	SYRGIRHRR	HLA-A*31:01, HLA-C*14:02	85-93	1.3	0.28	Non-Allergenicity
P3	LEIKEVHDL	HLA-B*40:01, HLA-B*44:02, HLA-B*40:02, HLA-B*18:01	36-44	0.9	0.002	Non-Allergenicity
	GMDRVQADY	HLA-A*01:01, HLA-A*29:02, HLA-C*08:02	67-75	0.6	0.03	Non-Allergenicity
	IRGRALRHL	HLA-B*14:02, HLA-C*07:02, HLA-C*07:01	115-123	0.6	0.15	Non-Allergenicity
	AEIEADAIL	HLA-B*40:01, HLA-B*40:02, HLA-B*44:02, HLA-B*18:01, HLA-B*39:01	151-159	0.8	0.31	Non-Allergenicity

**Table 3A:** Top five HTL epitopes selected for endocarditis vaccine.

Protein	Epitopes	Alleles	Position	Antigenicity	IL4	IL10	IFN-γ
P1	ISSYRGIRHRRGLPV	HLA-DRB1*15:06, HLA-DRB1*11:01	83-97	0.5	Inducer	Inducer	Positive
P2	YVGEFVRRKEGKTGK	HLA-DRB1*11:14, HLA-DRB1*13:23	164-178	1.0	Inducer	Inducer	Positive
P3	LMEIGSYRGIRHRRG	HLA-DRB1*11:01	80-94	0.7	Inducer	Inducer	Positive
	STDTTAALRAAEIEA	HLA-DQA1*04:01/DQB1*04:02, HLA-DQA1*03:01/DQB1*03:02	141-155	1.1	Inducer	Inducer	Positive
	LRIMDSTASTISMDN	HLA-DRB1*13:01, HLA-DRB1*04:10, HLA-DRB1*04:23, HLA-DRB1*01:02, HLA-DRB1*08:13, HLA-DRB1*11:02	195-209	0.7	Inducer	Inducer	Positive

**Table 3B:** Top seven LBL epitopes selected for endocarditis vaccine.

Protein	Epitopes	Score	Position	Antigenicity	Immunogenicity	Allergenicity
P1	RVVISLTYIYGIGTST	0.85	14	0.6	0.26	Non-Allergenicity
P2	IKMNIEGNEVTFTRPN	0.77	41	1.2	0.55	Non-Allergenicity
	DSKEMKTIHGTTRANF	0.86	57	1.0	0.03	Non-Allergenicity
	GVSEDVRVRELTNEQT	0.64	38	1.4	0.38	Non-Allergenicity
P3	RGIRHRRGLPVRGQNT	0.89	87	1.3	0.25	Non-Allergenicity
	SGEALAGERGVGIDIK	0.81	14	1.7	0.60	Non-Allergenicity
	ALEIKEVHDLGIEIAL	0.64	35	1.4	0.52	Non-Allergenicity

### 3.4 Epitopes population coverage

**Figure 1:**
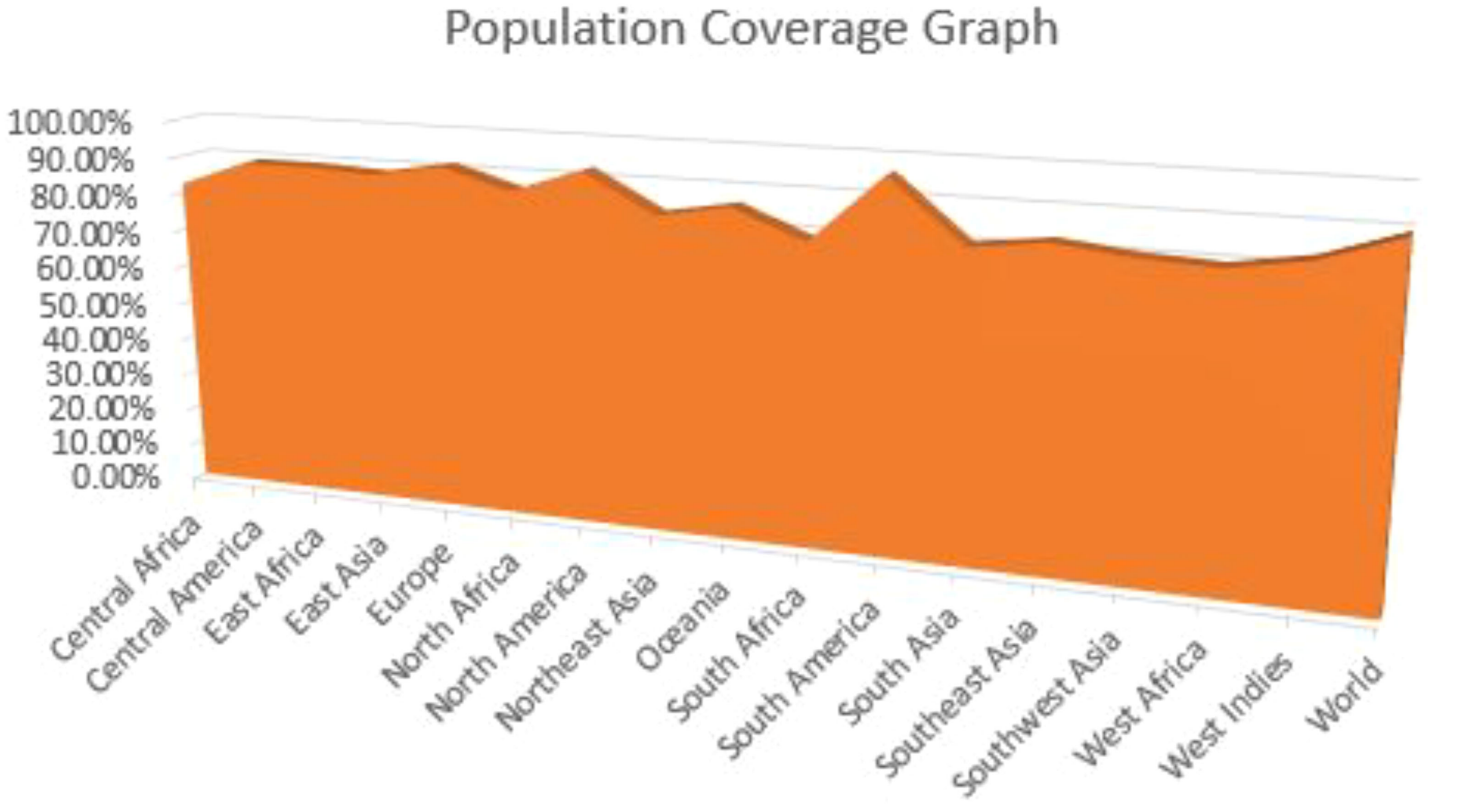
Graph of the world’s population coverage based on selected t cell epitopes.

The vaccine we have developed is expected to provide widespread coverage across a significant proportion of the human population. We employed the IEDB population coverage analysis tool to evaluate the extent to which the vaccine is effective in protecting the entire global human population. The inclusion of 54 HLA alleles for T-cell epitope prediction (**Figure 1**) achieved a population coverage of roughly 89.55% for the worldwide human population.

### 3.5 Multi-epitope vaccine construction

**Figure 2:**
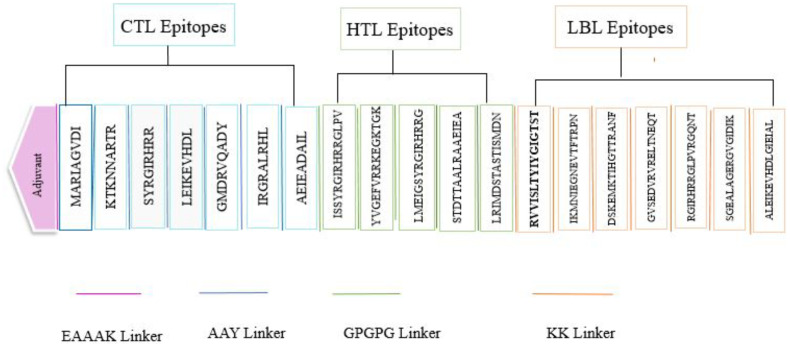
Graphical representation of the designed endocarditis vaccine.

The design of the multi-epitope vaccine comprised five linear B cell epitopes alongside twelve T cell epitopes, which including seven MHC-I and five MHC-II. The epitopes were interconnected through the use of KK, GPGPG, and AAY linkers. Each anticipated epitope was determined to be significantly antigenic, devoid of toxicity, and free from allergens following a meticulous selection process. An EAAAK linker was used to attach the 236 amino acid cholera enterotoxin subunit B to the vaccine’s N-terminus (**Figure 2**).

### 3.6 Post vaccine analysis

#### 3.6.1 Identification of physiochemical characteristics

The vaccine design, consisting of 436 amino acids, was analyzed using the ProtParam server and found to have a molecular weight of 47 kDa. The resulting design, possessing a molecular weight under 110 kDa, is suitable for vaccine use. The vaccine had forty-two residues that were positively charged, specifically Arginine and Lysine. The predicted half-life in yeast is 20 hours; in human reticulocytes, it is 1.9 hours, and in *E. coli*, it exceeds 10 hours. The final construct possesses the chemical formula C_2109_H_3439_N_633_O_616_S_13_ and consists of precisely 6810 atoms. The vaccine’s high solubility can be attributed to its robust water-binding interaction and polar nature, as evidenced by its aliphatic index of 81.10 and GRAVY value of -0.4602. The solubility probability of the projected vaccine structure was determined to be 0.845522. Furthermore, VaxiJen evaluated the multi-epitope vaccination for its potential toxicity, allergenicity, and antigenicity. The projected antigenicity at a bacterial model threshold of 0.5 was determined to be 0. 8591. The prospective vaccine underwent toxicity and allergenicity assessments to ensure its non-induction of adverse assessment of secondary reactions upon administration. Fortunately, the findings indicated that the vaccine candidate did not elicit any adverse side effects.

#### 3.6.2 Validation and prediction of vaccine structure (Secondary and Tertiary)

**Figure 3:**
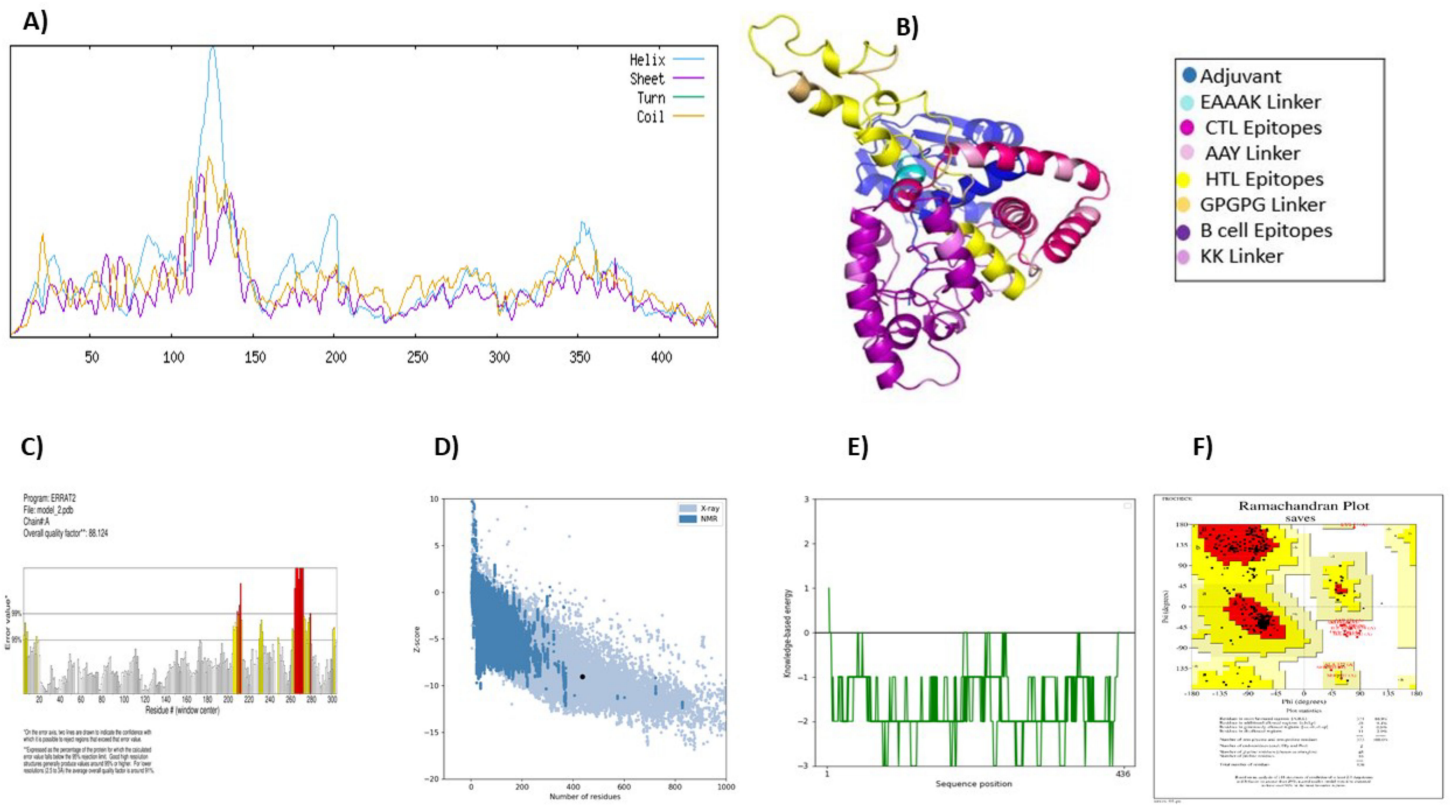
Vaccine structure prediction and validation. **(A)** secondary structure prediction using SOPMA, highlighting alpha helices, beta-sheets, and coils **(B)** 3D structural modeling performed by ROBETTA **(C)** quality assessment of the 3D model using ERRAT to identify structural errors **(D)** structural validation through ProSA-web for overall model evaluation **(E)** ProSA-web energy plot depicting the stability of the predicted structure **(F)** ramachandran plot analysis using PROCHECK to assess the stereochemical quality of the model.

The vaccine construct was subjected to a secondary structure analysis using the SOPMA server. The structural characteristics revealed that 41.74% (182 amino acids) of the structure was α-helix, 12.39% (54 amino acids) was β-strands, and 45.87%(200 amino acids) was random coils (**Figure 3A**).

The Robetta server was used to create the vaccine’s tertiary structure, yielding five projected structures for the query sequence. Each structure was designated with a confidence value of 0.45. After thorough evaluation, Model-1 was chosen for further study (**Figure 3B**). Independent evaluations of the quality and possible flaws in the basic 3D model was performed by ERRAT and ProSA-web server Based on the ERRAT analysis, the selected model showed an amazing 88.12% general quality factor (**Figure 3C**). In addition, the ProSA-web server measured the input vaccine’s Z score to be -9.04, suggesting its high-quality structure (**Figure 3D**). This was evident from the positive energy values observed in the main parts of the energy plot, such as the central and C-terminal regions of the vaccine construct (**Figure 3E**). The predicted three-dimensional structure of the vaccine construct was analyzed using the PROCHECK online service, employing a Ramachandran plot (**Figure 3F**). The research revealed that 86.9% of residues were situated in favored regions, whereas 9.4% were detected in allowed regions. Only a tiny fraction of residues (0.8%) were inside the generously permitted range, while 2.9% were identified as outliers. The collective findings from PROCHECK, ERRAT, and ProSA-web server provide highly compelling evidence for the outstanding quality of the 3D-modeled protein.

#### 3.6.3 Conformational B-cell epitopes prediction

The ElliPro server was implemented to anticipate potential conformational B-cell epitopes in the vaccine construct, emphasizing its tertiary structure and folding. Seven novel epitopes were identified in this analysis, with scores ranging from 0.509 to 0.834 (**Table 4**). Furthermore, seven discontinuous B-cell epitopes were identified, containing 161 residues. The scores for these epitopes varied from 0.53 to 0.912 (**Table 5**).

**Table 4:** Endocarditis vaccine’s linear/continuous b cell epitopes.

No.	Chain	Start	End	Peptide	Number of residues	Score
1	A	226	272	RGLPVGPGPGYVGEFVRRKEGKTGKGPGPGLMEIGSYRGIRHRRGGP	47	0.834
2	A	108	116	VWNNKTPHA	9	0.81
3	A	391	411	RGLPVRGQNTKKSGEALAGER	21	0.762
4	A	30	83	CAEYHNTQIYTLNDKIFSYTESLAGKREMAIITFKNGAIFQVEVPGSQHIDSQK	54	0.737
5	A	150	180	RAAYSYRGIRHRRAAYLEIKEVHDLAAYGMD	31	0.688
6	A	330	366	KIKMNIEGNEVTFTRPNKKDSKEMKTIHGTTRANFKK	37	0.67
7	A	309	313	DNKKR	5	0.509

**Table 5:** Endocarditis vaccine’s conformational/discontinuous b cell epitopes.

No.	Residues	Number of residues	Score
1	A:G157, A:I158, A:R159, A:R162	4	0.912
2	A:Y219, A:R220, A:G221, A:R226, A:G227, A:L228, A:P229, A:V230, A:G231, A:P232, A:G233, A:P234, A:G235, A:Y236, A:V237, A:G238, A:E239, A:F240, A:V241, A:R242, A:R243, A:K244, A:E245, A:G246, A:K247, A:T248, A:G249, A:K250, A:G251, A:P252, A:G253, A:P254, A:G255, A:L256, A:M257, A:E258, A:I259, A:G260, A:S261, A:Y262, A:R263, A:G264, A:I265, A:R266, A:H267, A:R269, A:G270, A:G271, A:P272	49	0.8
3	A:N25, A:T27, A:C30, A:A31, A:E32, A:Y33, A:H34, A:N35, A:T36, A:Q37, A:I38, A:Y39, A:T40, A:L41, A:N42, A:D43, A:K44, A:I45, A:F46, A:S47, A:Y48, A:T49, A:E50, A:S51, A:L52, A:A53, A:G54, A:K55, A:R56, A:E57, A:M58, A:A59, A:I60, A:I61, A:T62, A:F63, A:K64, A:N65, A:G66, A:A67, A:I68, A:F69, A:Q70, A:V71, A:E72, A:V73, A:P74, A:G75, A:S76, A:Q77, A:H78, A:I79, A:D80, A:Q82, A:K83, A:Y97, A:K102, A:V103, A:E104, A:V108, A:W109, A:N110, A:N111, A:K112, A:T113, A:P114, A:H115, A:A116, A:A118, A:A202, A:E203	71	0.711
4	A:A151, A:A152, A:Y153, A:S154, A:Y155, A:R156, A:H160, A:R161, A:A163, A:A164, A:Y165, A:L166, A:E167, A:I168, A:K169, A:H172, A:D173, A:L174, A:A175, A:A176, A:Y177, A:G178, A:M179, A:D180, A:Q183	25	0.655
5	A:M1, A:I2, A:K3, A:M308, A:K312, A:Y322, A:G323, A:I324, A:G325, A:T326, A:S327, A:K330, A:I331, A:K332, A:N334, A:I335, A:E336, A:G337, A:N338, A:E339, A:V340, A:T341, A:T343, A:R344, A:P345, A:N346, A:K347, A:K348, A:D349, A:S350, A:K351, A:E352, A:M353, A:K354, A:T355, A:I356, A:H357, A:G358, A:T359, A:T360, A:R361, A:A362, A:N363, A:K365, A:K366, A:G392, A:L393, A:P394, A:V395, A:R396, A:G397, A:Q398, A:N399, A:T400, A:K401, A:K402, A:S403, A:G404, A:E405, A:A406, A:L407, A:A408, A:G409, A:E410, A:R411, A:V413, A:G414, A:I415, A:D416, A:I417, A:K418, A:K419, A:I432, A:E433, A:I434, A:A435, A:L436	77	0.643
6	A:G273, A:L377, A:T378, A:N379, A:E380, A:Q381, A:K384, A:I387, A:R388, A:R391	10	0.555
7	A:D309, A:N310, A:K311	3	0.53

### 3.7 Disulfide engineering

**Figure 4:**
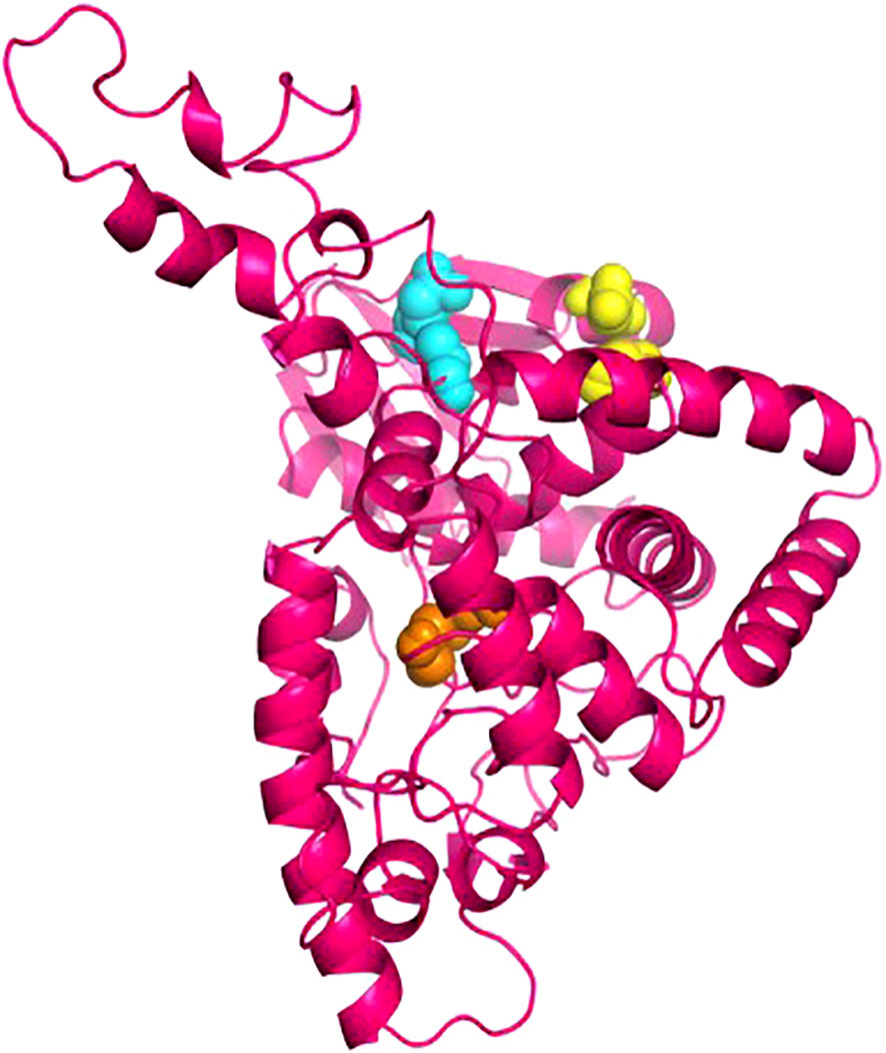
The disulphide engineering of the vaccine construct. This figure illustrates the introduction of disulphide bonds to enhance the structural stability of the vaccine construct. Three selected mutant pairs, identified based onX^3^ value and energy are highlighted using yellow, cyan, and orange color spheres, representing their positions within the 3D structure.

The Disulfide by Design 2.13 server was employed to analyze the vaccine sequence, and a total of thirty residue pairs that could potentially produce disulfide bonds were identified. By considering the X3 parameters and bond energy, three pairs of residues (23PRO-28ASP, 104GLU-123ALA, and 210LEU-303ALA) were selected based on their findings meeting the predefined requirements (**Figure 4**).

### 3.8 Immune simulation

**Figure 5:**
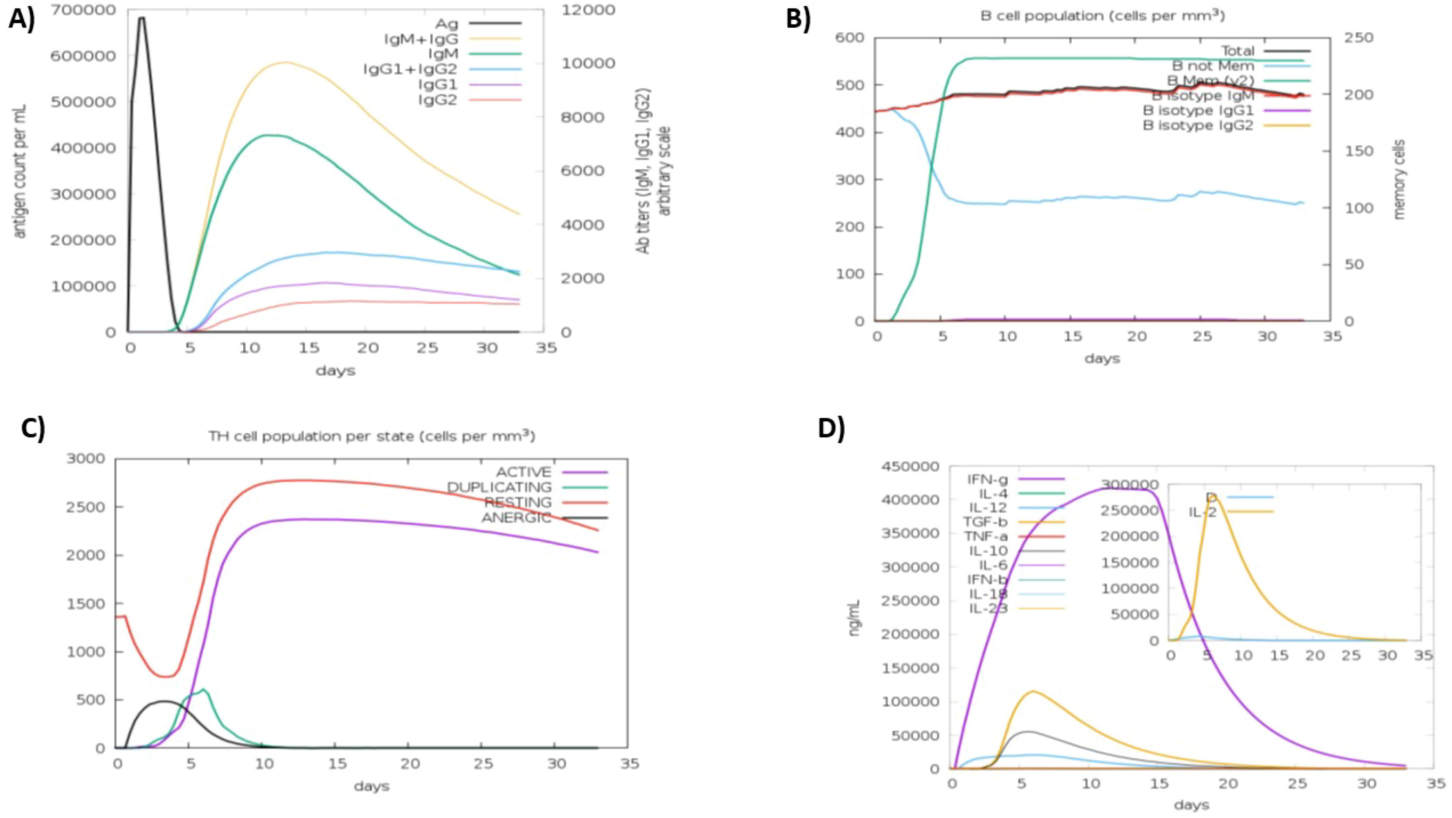
Anticipated immune response after vaccine administration **(A)** immunoglobulin response, showing antigen interaction and isotype specific immunoglobulin distribution. **(B)** b-lymphocytes response, including total memory cell count and subdivision into IgM, IgG1, and IgG2 isotypes **(C)** CD4+ t-helper lymphocyte dynamics, categorized by functional states (i-e active, anergic, resting, duplicating) **(D)** interleukins and cytokines concentration, reflecting the immune signaling response, with the insert plot representing the danger signal activation.

The C-ImmSim server was used to increase the half-life of immune cells in order to study their robust and efficient immune responses. This technique demonstrated that some cells had a significantly longer half-life than others, resulting in their prolonged survival. The immune simulation outcomes generated by the C-ImmSim server aligned closely with empirical immunological responses observed in real-world scenarios. The response was primarily defined by elevated levels of IgM. Furthermore, there was a rise in the expression of several immunoglobulins, including IgG1+IgG2, IgM, and IgG+IgM, leading to a reduction in serum antigen levels (**Figure 5A**). This rise in B-cell population was followed by memory extension (**Figure 5B**) and a substantial increase in the number of Th cells (**Figure 5C**). An increase in IFN-γ production was also observed as a result of vaccination (**Figure 5D**). The results pertaining to the T cell population closely aligned with the expected patterns as memory evolved, whereas the other immune cell populations exhibited a degree of stability.

### 3.9 Molecular docking

MHC molecules and toll-like receptors are examples of immunological receptors that a vaccine must attach to firmly in order to successfully elicit a potent immune response. Using HADDOCK version 2.4, the relationships between vaccines and human TLR4, MHCI, and MHCII were investigated. The vaccine binding scores to TLR4, MHCI, and MHCII were 70.5 +/- 51.0 kcal/mol, 97.3 +/- 12.5 kcal/mol, and -133.9 +/- 22.5 kcal/mol, respectively. These values were within the range of the standard deviation. Data about docking is presented in **Table 6**.

**Table 6:** Docking statistics of vaccine with immune receptors.

Docking Statistics	TLR4	MHC-I	MHC-II
HADDOCK score	70.5 +/- 51.0	97.3 +/- 12.5	-133.9 +/- 22.5
Cluster size	4	7	22
RMSD from the overall lowest-energy structure	1.6 +/- 1.0	24.9 +/- 0.1	1.3 +/- 0.8
Van der Waals energy	-68.1 +/- 9.2	-87.4 +/- 9.4	-99.2 +/- 14.7
Electrostatic energy	-719.3 +/- 180.0	-455.8 +/- 18.8	-601.7 +/- 75.1
Desolvation energy	31.7 +/- 11.2	12.0 +/- 3.3	1.6 +/- 9.4
Restraints violation energy	2507.4 +/- 343.8	2638.5 +/- 110.3	840.7 +/- 89.4
Buried Surface Area	3532.0 +/- 244.2	2793.2 +/- 171.1	3812.5 +/- 192.7
Z-Score	-1.3	-1.0	-2.1

**Figure 6:**
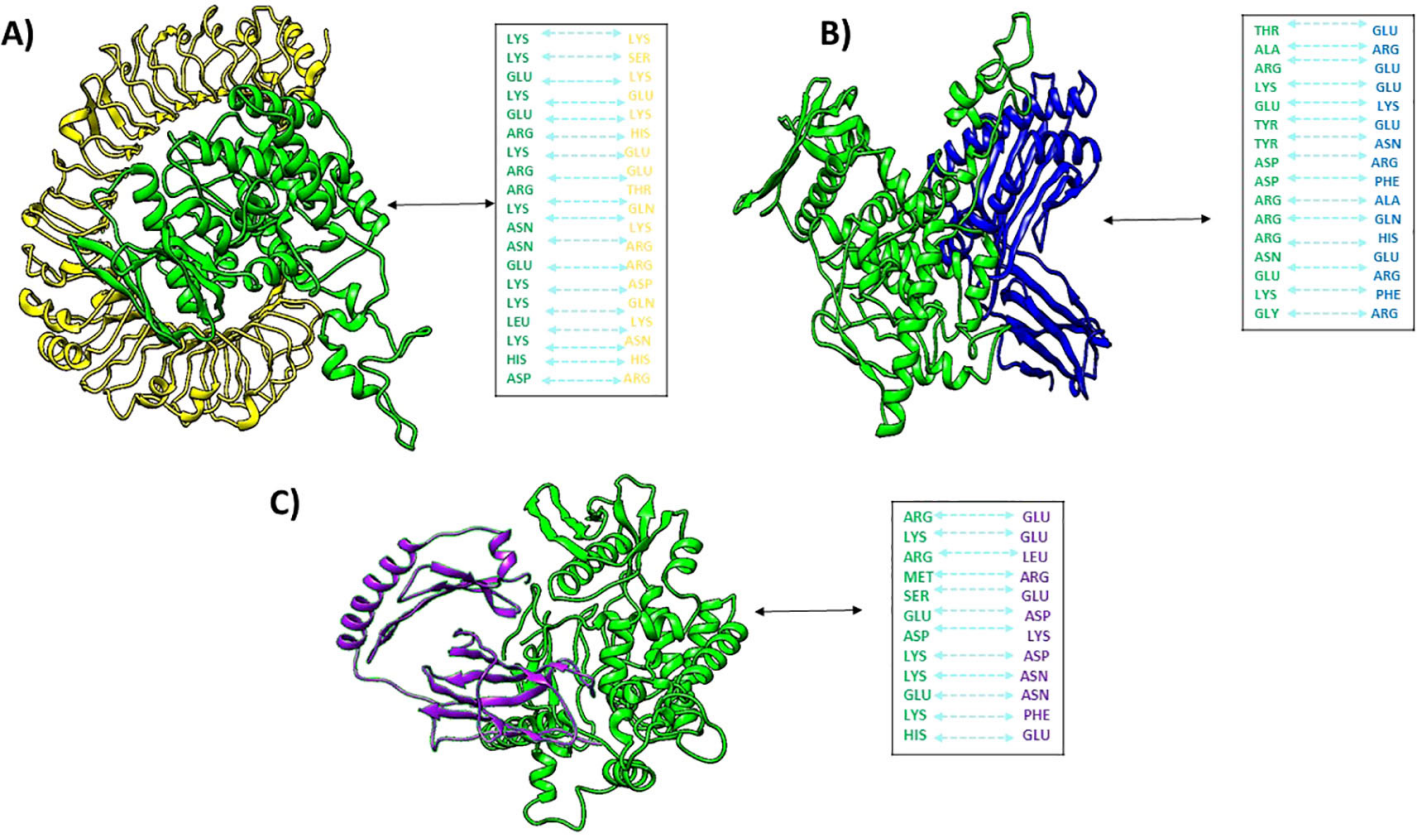
Docked complexes of **(A)** vaccine (green) with human tlr4 (yellow), **(B)** vaccine with MHCI (blue), and **(C)** vaccine with MHCII (purple), along with their hydrogen bond interactions.

**Figures 6A–C** depicts docked complexes with the vaccine (in green) and receptor molecule (in yellow), as well as MHCI (in blue) and MHCII (in purple), respectively. The PDBsum server was utilized in order to produce a map of connections between docked complexes as well as discoveries regarding the binding interactions that occur between vaccine components and receptor molecules. As part of the research, a schematic depiction of the interactions between docked proteins was presented. These interactions included both H-bond and non-bonded combinations. Upon analysis, it was discovered that the vaccine and TLR4, MHCI, and MHCII possessed 24, 23, and 14 hydrogen bonding interactions, respectively, with a range of 3.11 Å, 3.23 Å, and 3.27 Å.

### 3.10 MD simulation

#### 3.10.1 MHC-I

**Figure 7:**
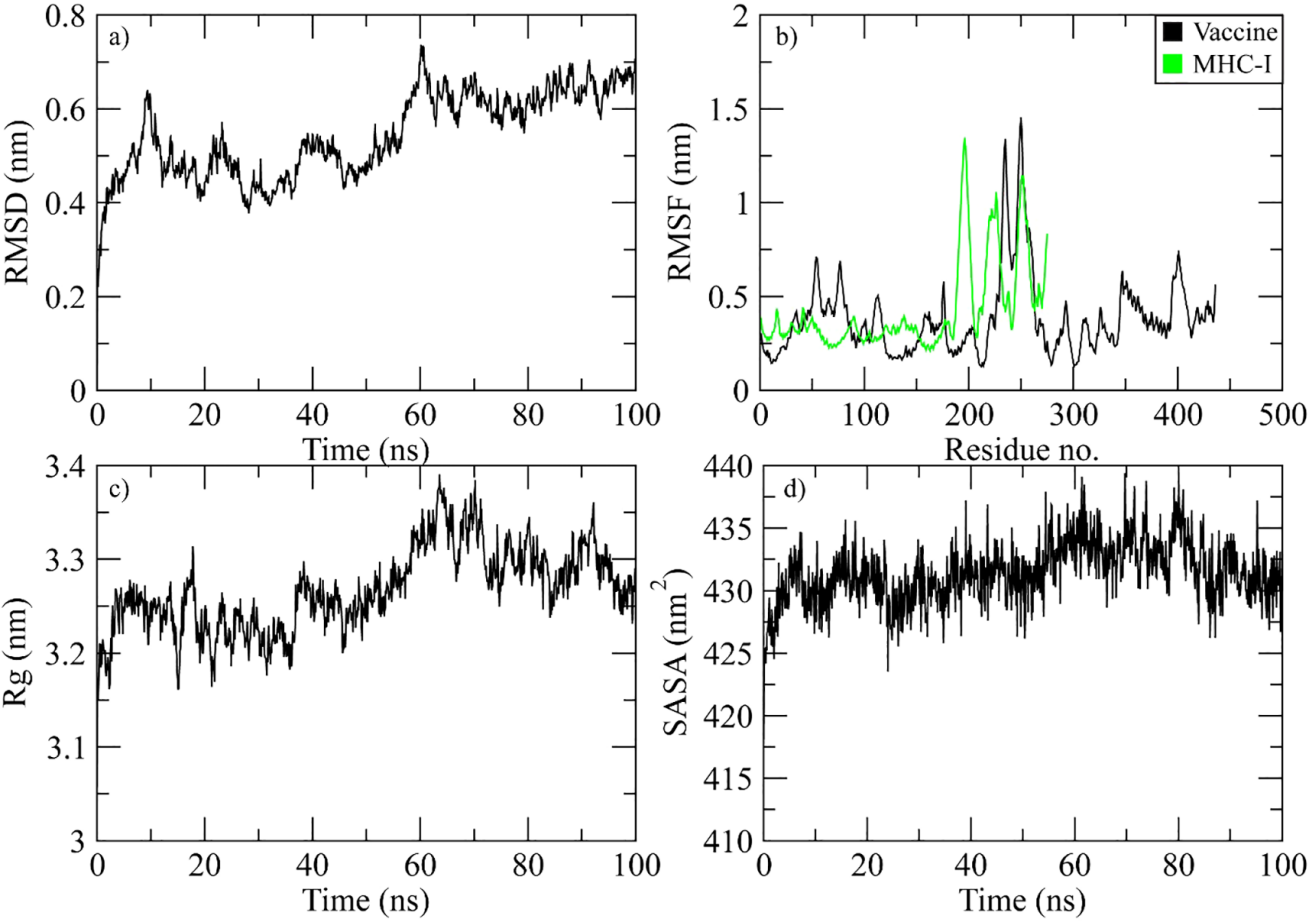
Simulation analysis of the MHC-I complex. **(a)** the RMSD of Cα atoms of vaccine indicating structural stability over time. **(b)** residue flexibility analysis showing the dynamic behavior of vaccine and MHC I- residues. **(c)** radius of gyration assessing the compactness of the complex. **(d)** The SASA plot evaluating the exposure of the complex to the solvent environment.

The RMSD of the Cα atoms in the protein was used to identify the stability of the complex during the simulation. The complex’s RMSD differed by 0.6 nm at 10 ns and then decreased by 0.2 nm every 20 to 40 ns. After 40 ns, the RMSD gradually decreased to 0.7 nm, and it maintained a range of 0.6-0.7 nm towards the end of simulation (**Figure 7a**). To study protein residue flexibility, we measured and plotted root mean square fluctuation (**Figure 7b**). Residues with higher RMSF value signify the flexible residues while lower value denote rigidity. RMSF plot reveals the residues of vaccine ranging from 50 to 70, 230 to 280, and 390 to 410 displayed significant fluctuations while the residues of MHC-I from the c-terminal showed high flexibility during simulation. Similarly, the radius of gyration (Rg) was calculated throughout the simulation to assess the compactness of the vaccine complex. Increased Rg values indicate that the protein experienced unfolding events during the simulation. **Figure 7c** shows the behavior of Rg during simulation. The plot revealed that Rg value demonstrated stability till 60 ns in the range of 3.25 nm and then increased to 3.4 nm at 70 ns. However, the Rg values attained the initial range at the end of simulation. Additionally, the solvent accessible surface area of the complex was measured. The SASA plot revealed that the SASA values of the complex remained in the range of 430 to 435 nm^2^ throughout the simulation (**Figure 7d**).

#### 3.10.2 MHC-II

**Figure 8:**
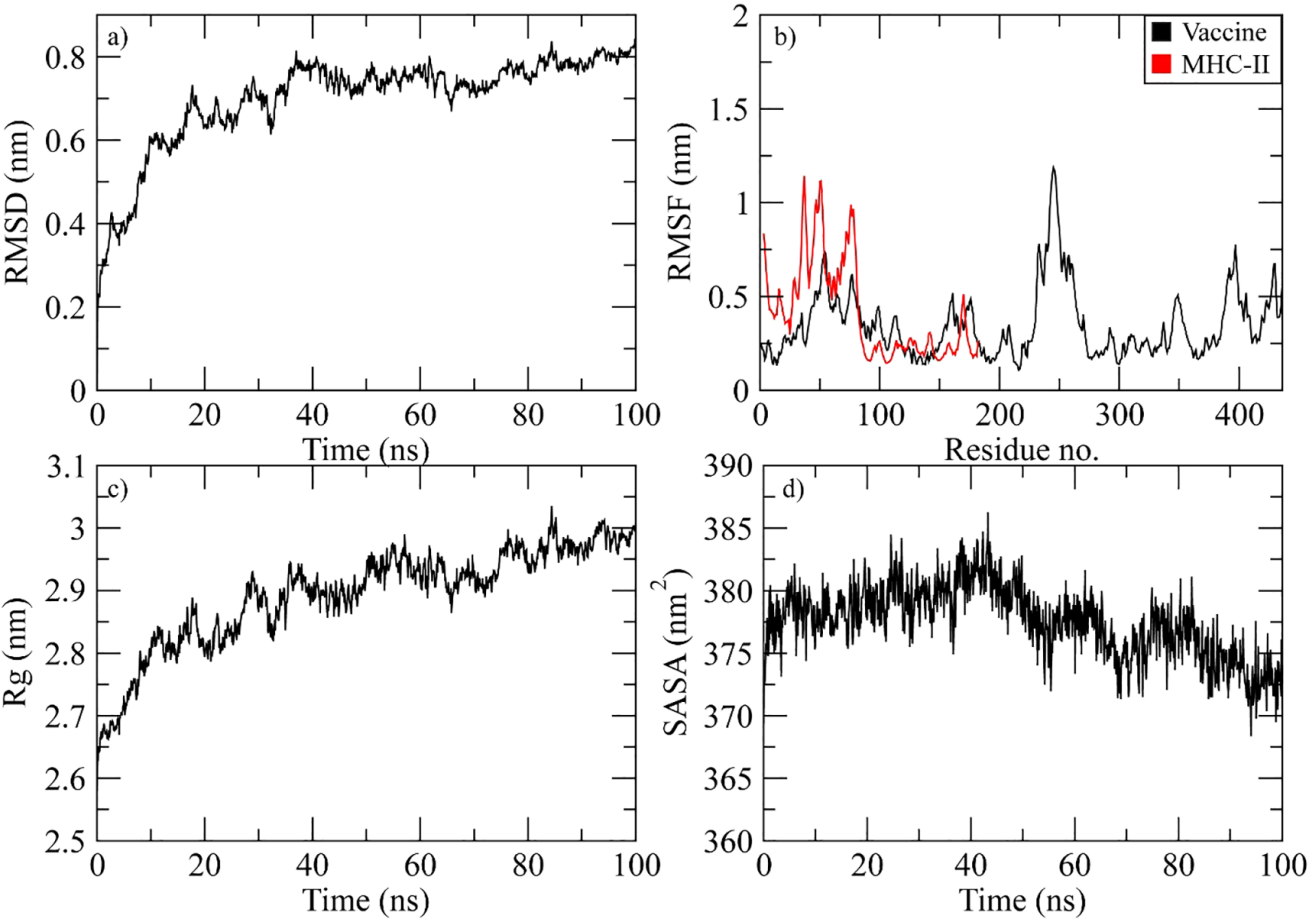
Simulation analysis of the MHC-II complex. **(a)** The RMSD of Cα atoms of vaccine indicating structural stability over time. **(b)** Residue flexibility analysis showing the dynamic behavior of vaccine and MHC II- residues. **(c)** Radius of gyration assessing the compactness of the complex. **(d)** The SASA plot evaluating the exposure of the complex to the solvent environment.

The complex stability was determined during the simulation by calculating the RMSD of the Cα atoms of the protein. The RMSD of complex gradually increased to 0.8 nm at 40 ns and then attained stability in this range till the end of simulation (**Figure 8a**). The RMSF plot of the complex revealed that the residues of vaccine ranging from 50 to 70, 230 to 280, and 390 to 410 displayed significant fluctuations while the residues of MHC-II from the N-terminal showed high flexibility during simulation (**Figure 8b**). The Rg plot of the vaccine-MHC-II complex revealed that Rg values gradually increased to 2.9 nm at 40 ns and the maintained the range of 2.9-3 nm till the end of simulation (**Figure 8c**). The SASA plot of vaccine-MHC-II complex revealed that the SASA values of the complex remained in the range of 375 to 380 nm^2^ throughout the simulation (**Figure 8d**).

#### 3.10.3 TLR4

**Figure 9:**
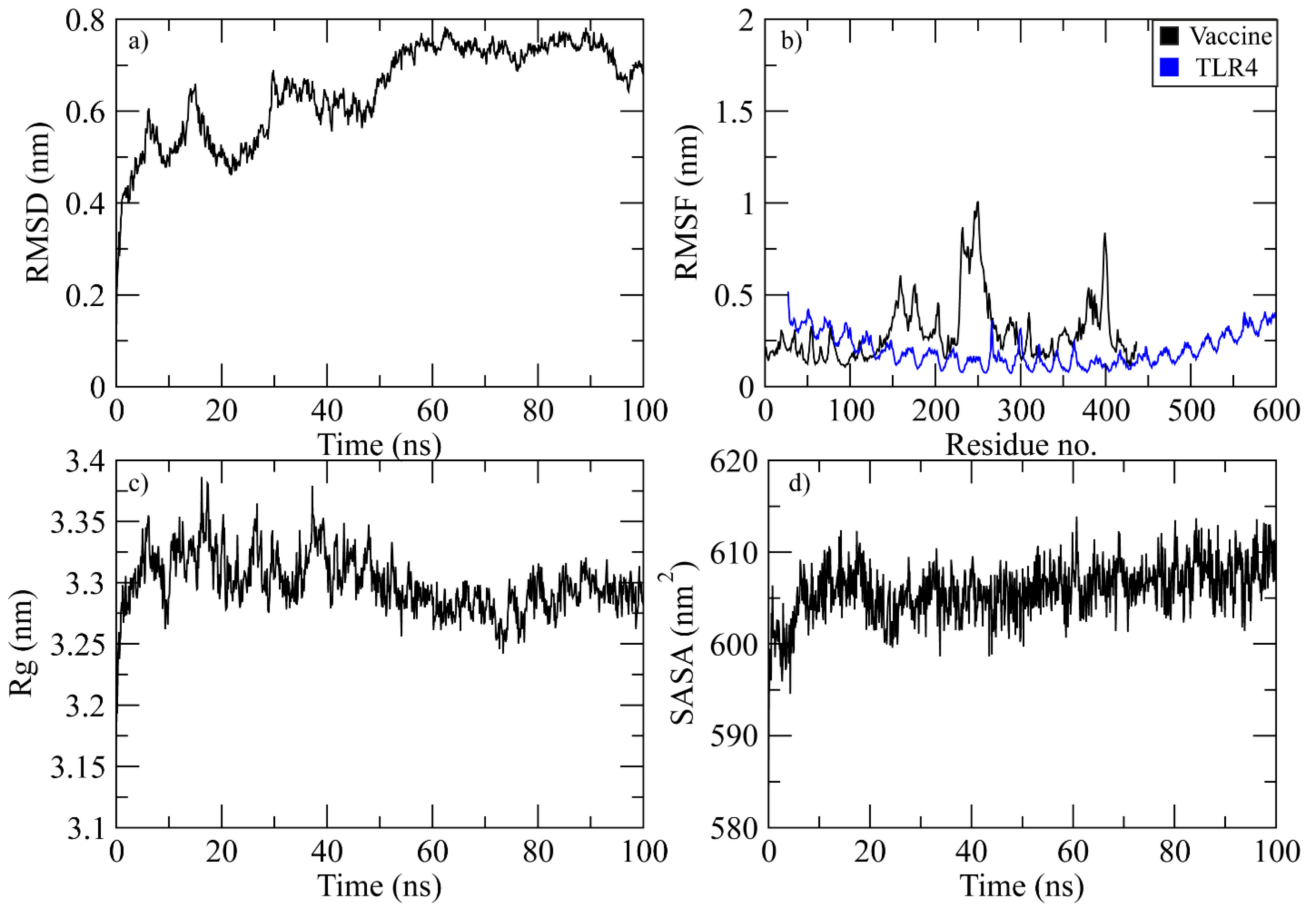
Simulation analysis of the TLR4 complex. **(a)** The RMSD of Cα atoms of vaccine indicating structural stability over time. **(b)** Residue flexibility analysis showing the dynamic behavior of vaccine and TLR4- residues. **(c)** Radius of gyration assessing the compactness of the complex. **(d)** The SASA plot evaluating the exposure of the complex to the solvent environment.

At 40 ns, the RMSD of the vaccine-TLR4 complex was between 0.4 and 0.6 nm. After that, it started to rise and reached 0.75 nm at 60 ns. It stayed in this range until 95 ns, when it dropped to 0.7 nm (**Figure 9a**). The RMSF plot of the complex revealed that the residues of vaccine ranging from 150 to 180, 230 to 280, and 390 to 410 displayed significant fluctuations while the TLR4 residues did not show fluctuations during simulation (**Figure 9b**). The Rg plot of the vaccine-TLR4 complex revealed that Rg values remained in the range of 3.3-3.35 nm throughout the simulation (**Figure 9c**). The SASA plot of vaccine-TLR4 complex revealed that the SASA values of the complex remained in the range of 600 to 610 nm^2^ throughout the simulation (**Figure 9d**).

### 3.11 *In silico* cloning

**Figure 10:**
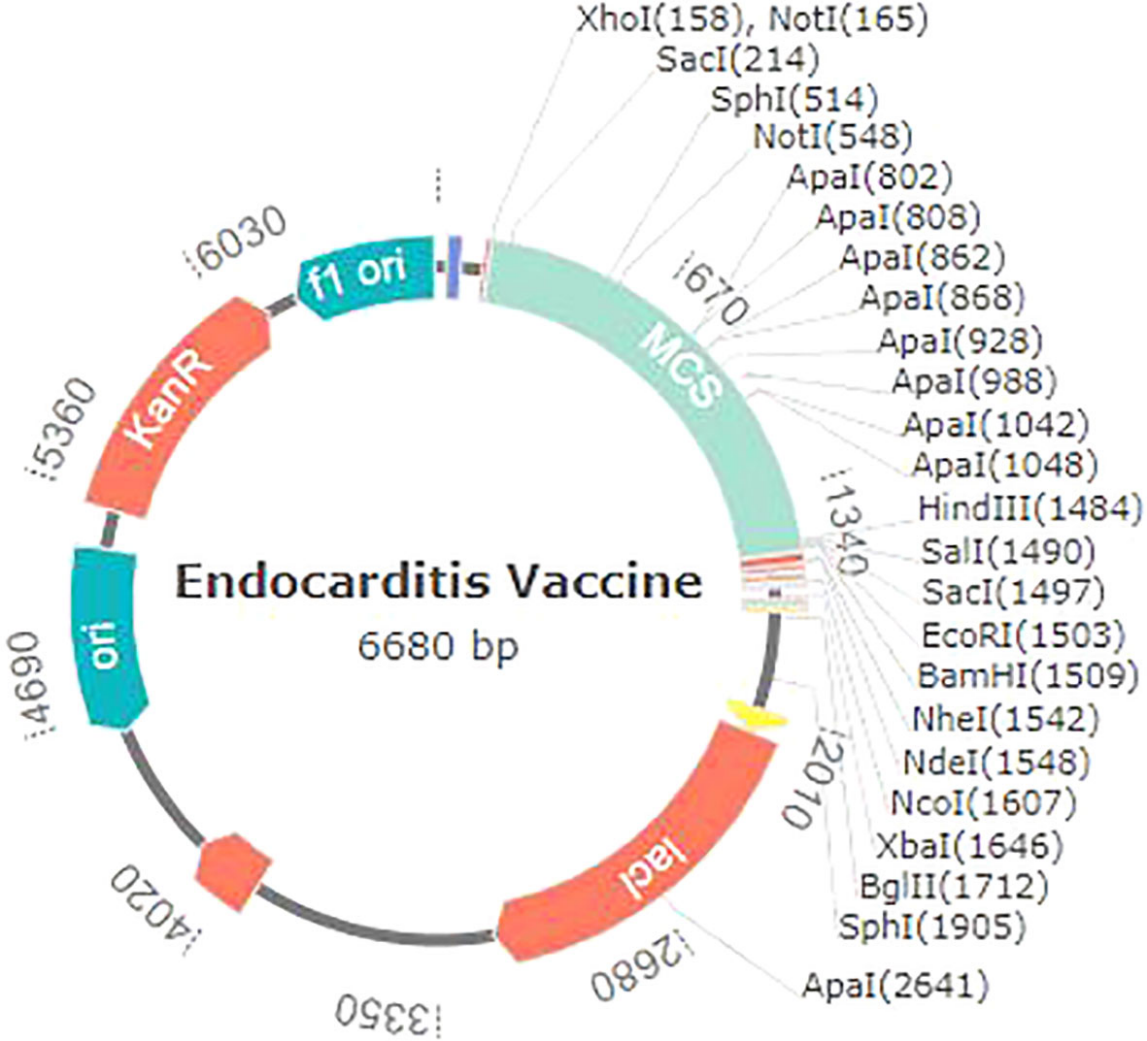
Endocarditis vaccine *in silico* restriction cloning with the pet28a (+) expression vector.

Different codon usage patterns prevent foreign genes from being translated, hence the only effective technique to boost translational efficacy is codon adaptation. Using vectorBuilder to optimize codon usage, we were able to successfully express the vaccine we created against the *E. coli* K12 strain in the *E. coli* host. The vaccine exhibits a GC content of 49.77% and a CAI of 0.94%, which substantiates this assertion. Subsequently, the altered codon sequence of the proposed vaccine was integrated into the PET28a (+) *E. coli* expression vector within the segment located between the BamHI and XhoI restriction sites (**Figure 10**).

## 4 Discussion

Endocarditis is a dangerous infection of the heart’s internal lining that typically affects the heart’s valves. Cardiac valve disease is mostly caused by bacterial pathogens, a significant number of which develop biofilms on heart valves, rendering them resistant to antibiotic therapies. Given the growing resistance of these bacteria to traditional antibiotics and the intricate nature of biofilm production, it is imperative to find other therapeutic approaches, such as vaccinations, to avoid the onset of endocarditis. Developing a successful vaccination against endocarditis-associated infections is difficult because of the wide range of bacterial species, their characteristic virulence factors, and their capacity to elude the host’s immunological response (17). Integrating core proteomics can effectively tackle these issues by offering a valuable understanding of the molecular mechanisms of these diseases, enabling the discovery of conserved protein antigens essential for bacteria’s survival and pathogenicity.

The present study utilized core proteome analysis and the computationally based reverse vaccinology approach to forecast likely antigenic proteins and identify potential vaccine candidates from endocarditis-related pathogens. The core proteome analysis included 121 highly pathogenic strains of ten different pathogens. A subtractive proteomics workflow was applied to the core proteome to identify vaccine candidates that were virulent, non-homologous, antigenic, and non-allergenic. Three proteins have been identified as potential targets for vaccine development: 30S ribosomal protein S13, 50S ribosomal protein L6, and UMP Kinase.

Target proteins were expected to have HTL, CTL, and LBL epitopes. Based on the multi-epitope candidate’s immunogenicity, antigenicity, allergenicity, and toxicity, epitopes were selected. Vaccine expression, stability, and folding can be improved using linkers. This vaccine structure was formed by using AAY, KK, and GPGPG linkers to connect the CTL, LBL, and HTL epitopes, as shown in numerous prior investigations (69–73). Additives must be used in multi-epitope vaccines since they are not capable of producing adequate immunogenicity on their own (74). As an adjuvant, cholera toxin B subunit (CTB) was used. Prior studies have thoroughly examined the application of CTB as a traditional mucosal adjuvant to augment the immunogenicity of vaccines (71, 75). The adjuvant function was executed by utilizing the EAAAK linker. Prior investigations have shown that protein domains can undergo independent folding and function as a result of the rigid structure of this a-helical linker. This linker can greatly enhance the stability of the fusion proteins, especially in high-temperature conditions, which is crucial for vaccines (76). The final vaccine contained 436 amino acids. However, larger vaccines were found in other investigations. Hence, we think it will be reliable and interpretable.

In order to be efficacious, a vaccine molecule must provide extensive protection against a wide range of global populations. An essential step in developing a vaccine based on HLA genotype frequencies is to ascertain the population proportions in the targeted endemic regions. Based on the data, the chosen CTL and HTL epitopes covered 89.55% of the global population.

The suggested vaccine is advantageous since its sufficient molecular weight makes purification easier. Moreover, the predicted results show that the endocarditis vaccine is safe and effective without causing allergic reactions. Apart from including antigens that cause the immune system of the host to react, a good vaccine should also be able to create long-lasting adaptive immunity. The delivery of the vaccine within a fluidic environment of the host organism necessitates that solubility be regarded as a critical characteristic throughout the development process. Consequently, the vaccine’s solubility was assessed and determined to be highly soluble.

The spatial arrangement of important protein constituents and the study of protein dynamics, function, and ligand-protein interactions are facilitated by the three-dimensional structure, which provides valuable insights (77, 78). Enhanced improvement of the vaccine greatly enhanced its favorable characteristics. Refining the vaccine structure greatly enhanced its desirable characteristics. A Ramachandran plot reveals that only a small number of residues are located in the prohibited area, whereas the majority are found in the preferred regions (86.9%). The Z-score and ERRAT quality factor for our vaccine results were -9.04 and 88.12%, respectively. A model with a quality factor exceeding 50% is deemed to possess good quality. The quality metric and z-score confirm the exceptional quality of our model. The vaccine construct was employed to predict B-cell epitopes in order to determine whether it contained an adequate number of epitopes for antibodies to recognize and bind to (79).

Efficacious delivery of a candidate vaccine throughout the host body requires a robust interaction between immunological receptors (such as TLR4, MHCI, and MHCII) and the vaccine. Furthermore, the molecular docking investigations and molecular dynamics modelling not only validated the strong contacts between the vaccine and TLR4, MHCI, and MHCII, but also demonstrated that a little energy expenditure was necessary for this sustained binding. During the protein-protein docking of the vaccine-TLR4, vaccine-MHCI, and vaccine-MHCII, minimal oscillations were observed in molecular dynamics simulations, while multiple hydrogen bonds were detected. The evidence compellingly indicates that the vaccine possesses the ability to effectively bind to immunological receptors.

Next, an immunological simulation was run on the C-IMMSIM server to assess the vaccine’s capacity to elicit an immune response. Nonetheless, the findings revealed a noteworthy improvement in memory T and B cells. Following the administration of the third dosage of the vaccine, a significant rise in the titer of IFN-γ was observed, accompanied by a modest increase in IL-2 levels. Nonetheless, the antibody levels were relatively diminished during the primary immune response when compared to the tertiary and secondary immunological reactions. The findings indicate that the proposed multi-epitope vaccination has the potential to effectively elicit an immune response, thereby fostering a strong immunity against viral infections.

Ultimately, the vaccine MRNA was amplified using the JCAT to ensure the effective translation of a multi-epitope vaccine by a specific expression method. To clone the expression vector pET28a (+), a modified DNA sequence was inserted between the cleavage sites of the BamHI and XhoI restriction enzymes. The GC content of 53.63% and CAI of 0.98 suggest that bacteria possess the capacity for producing proteins at a high level.

In summary, the development of a multi-epitope vaccine targeting endocarditis-associated pathogens using immuno-informatics and subtractive proteomics shows great potential and is an innovative research approach. This study showcases the feasibility of integrating advanced bioinformatics tools with conventional vaccine development approaches to create a highly efficient and safe vaccine against difficult diseases. Future research should concentrate on the empirical validation and improvement of the vaccine design, including preclinical *in vivo* experiments to evaluate the vaccine’s immunogenicity and protective effectiveness. A suitable animal model must be immunized, and immunological responses must be assessed by cytokine profiling, ELISpot or flow cytometry for T-cell activation, and ELISA for antibody titers. Challenge experiments should also be carried out to measure survival rates, and clinical symptoms after exposure in order to ascertain the vaccine’s potential for protection. These actions will facilitate the advancement towards clinical trials and the ultimate implementation in healthcare settings.

## 5 Conclusion

The utilization of immunoinformatic analysis and the development of multi-epitope vaccines targeting endocarditis-related microorganisms offer a hopeful strategy for combating the increasing danger of endocarditis, particularly in light of antibiotic resistance and limited vaccination alternatives. This study successfully created a vaccine that can effectively target numerous species at the same time. The vaccine achieves this by discovering and utilizing highly conserved and immunogenic epitopes across different pathogens, with the help of bioinformatics tools. This method not only improves the immune system’s capacity to identify and respond to a variety of pathogens, but it also has the potential to reduce immune evasion. Additionally, the *in silico* validation of vaccine candidates guarantees that the selected epitopes elicit robust and targeted immune responses, thereby enhancing the probability of effective clinical outcomes. Future research and clinical trials are essential for applying these computer-based discoveries to real-world situations. Nonetheless, the application of immunoinformatics in the design of vaccines holds considerable promise for the prevention of endocarditis and the improvement of public health outcomes.

## Data Availability

The original contributions presented in the study are included in the article/**Supplementary Material**. Further inquiries can be directed to the corresponding author/s.

## Supplementary material

Additional File 1 Complete list of bacterial strains used in this study.
