# Supplementary material for: Pan-vaccinomics strategy for developing a universal multi-epitope vaccine against endocarditis-related pathogens
Source: Front Immunol. 2025 Apr 11;16:1524128. doi: 10.3389/fimmu.2025.1524128 (PMC12021834; doi:10.3389/fimmu.2025.1524128)
Supplement: Additional File 1 — Complete list of bacterial strains used in this study. [file Table1.docx]

**List of pathogenic bacterial strain used in this study**

| ***Streptococcus mutans*** | | |
| --- | --- | --- |
|  | *Streptococcus mutans*  strain LAR01 | GCA_002995555.1 |
|  | *Streptococcus mutans*  strain MD | GCA_008831325.1 |
|  | *Streptococcus mutans*  strain NCH105 | GCA_009738105.1 |
|  | *Streptococcus mutans*  strain NCTC10832 | GCA_900636835.1 |
|  | *Streptococcus mutans*  strain P1 | GCA_011765545.1 |
|  | *Streptococcus mutans*  strain P6 | GCA_011765525.1 |
|  | *Streptococcus mutans*  strain S1 | GCA_011765505.1 |
|  | *Streptococcus mutans*  strain S4 | GCA_011765485.1 |
|  | *Streptococcus mutans*  B04Sm5 | GCA_014621675.1 |
|  | *Streptococcus mutans*  strain T8 | GCA_008831345.1 |
|  | *Streptococcus mutans*  strain UA140 | GCA_008831365.1 |
|  | *Streptococcus mutans*  UA159 | GCA_000007465.2 |
|  | *Streptococcus mutans*  UA159-FR | GCA_000817065.1 |
|  | *Streptococcus mutans*  COCC33-14 | GCA_024762195.1 |
|  | *Streptococcus mutans*  COCC33-14R | GCA_024762175.1 |
|  | *Streptococcus mutans*  strain LAB761 | GCA_003691695.1 |
| ***Streptococcus viridans*** | | |
|  | Streptococcus viridans strain NCTC3166 | GCA_900636365.1 |
| ***Streptococcus pyogenes*** | | |
|  | Streptococcus pyogenes M1 476 | GCA_000349925.2 |
|  | Streptococcus pyogenes A20 | GCA_000307535.1 |
|  | Streptococcus pyogenes M1 GAS | GCA_000006785.2 |
|  | Streptococcus pyogenes MGAS315 | GCA_000007425.1 |
|  | Streptococcus pyogenes NCTC12064 | GCA_900475035.1 |
| ***Staphylococcus aureus*** | | |
|  | USA300-ISMMS1 | GCA_000568455.1 |
|  | VC40 | GCA_000245495.1 |
|  | Newman | GCA_000010465.1 |
|  | COL | GCA_000012045.1 |
|  | ATCC_BAA1680_ | GCA_000815125.1 |
|  | USA300_FPR3757 | GCA_000013465.1 |
|  | 2395_USA500 | GCA_000746505.1 |
|  | DSM_20231 | GCA_001027105.1 |
|  | CA15 | GCA_001021895.1 |
|  | Z172 | GCA_001021895.1 |
|  | T0131 | GCA_000204665.1 |
|  | Gv69 | GCA_000769575.1 |
|  | TW20 | GCA_000027045.1 |
|  | Bmb9393 | GCA_000418345.1 |
|  | M121 | GCA_001021875.1 |
|  | USA300_TCH1516 | GCA_000017085.1 |
|  | NCTC_8325 | GCA_000013425.1 |
|  | 502A_RN6607 | GCA_000597965.1 |
|  | ECT-R_2 | GCA_000253135.1 |
|  | Mu50 | GCA_000009665.1 |
|  | Mu3 | GCA_000010445.1 |
|  | ED98 | GCA_000024585.1 |
|  | 04-02981 | GCA_000025145.2 |
|  | N315 | GCA_000009645.1 |
|  | FCFHV36 | GCA_000969225.1 |
|  | JH9 | GCA_000016805.1 |
|  | CN1 | GCA_000463055.1 |
|  | 11819-97 | GCA_000239235.1 |
|  | H-EMRSA-15 | GCA_000695215.1 |
|  | HO_5096_0412 | GCA_000284535.1 |
|  | RKI4 | GCA_001027045.1 |
|  | ST772-MRSA-V_DAR4145 | GCA_000828035.1 |
|  | MSSA476 | GCA_000011525.1 |
|  | MW2 | GCA_000011265.1 |
|  | 08BA02176 | GCA_000296595.1 |
|  | ST398_S0385 | GCA_000009585.1 |
|  | 6850 | GCA_000462955.1 |
|  | SA268 | GCA_000737615.1 |
|  | SA957 | GCA_000470845.1 |
|  | M013 | GCA_000237125.3 |
|  | SA40 | GCA_000470865.1 |
|  | JKD6159 | GCA_000144955.2 |
|  | RF122 | GCA_000009005.1 |
|  | CA-347 | GCA_000412775.1 |
|  | FORC_001 | GCA_000772025.1 |
|  | TCH60_MRSA_TCH60 | GCA_000159535.2 |
|  | ATCC_25923 | GCA_000756205.1 |
|  | MRSA252 | GCA_000011505.1 |
|  | ED133 | GCA_000210315.1 |
|  | LGA251 | GCA_000237265.1 |
| ***Enterococcus faecalis*** | | |
|  | Enterococcus faecalis R712 | GCA_009662495.1 |
|  | Enterococcus faecalis V583 | GCA_000007785.1 |
|  | Enterococcus faecalis D32 | GCA_000281195.1 |
|  | Enterococcus faecalis Symbioflor 1 | GCA_000317915.1 |
|  | Enterococcus faecalis OG1RF | GCA_000172575.2 |
| ***Streptococcus agalactiae*** | | |
|  | *S. agalactiae* GD201008-001 | GCA_000299135.1 |
|  | *S. agalactiae* SA20-06 | GCA_000302475.3 |
|  | *S. agalactiae* A909 | GCA_000012705.1 |
|  | *S. agalactiae* NEM316 | GCA_000196055.1 |
|  | *S. agalactiae* 2603 V/R | GCA_000007265.1 |
|  | *S. agalactiae*  GBS1-NY | GCA_000831145.1 |
|  | *S. agalactiae*  GBS6 | GCA_000831105.1 |
|  | *S. agalactiae* NCTC8184 | GCA_900636375.1 |
|  | *S. agalactiae*  HN016 | GCA_001190805.1 |
|  | *S. agalactiae*  HU-GS5823 | GCA_003966545.1 |
|  | *S. agalactiae*  NGBS 061 | GCA_000730215.2 |
|  | *S. agalactiae* BM110 | GCA_900155855.1 |
|  | *S. agalactiae* SG-M8 | GCA_002197325.1 |
|  | *S. agalactiae* SG-M1 | GCA_001275545.2 |
|  | *S. agalactiae* CJB111 | GCA_015221735.2 |
| ***Gemella morbillorum*** | | |
|  | *Gemella morbillorum*  FDAARGOS_741 | GCA_009730315.1 |
|  | *Gemella morbillorum* FDAARGOS_1501 | GCA_020097435.1 |
|  | *Gemella morbillorum* NCTC11323 | GCA_900476045.1 |
| ***Streptococcus pneumonia*** | | |
|  | Streptococcus pneumoniae R6 | GCA_000007045.1 |
|  | *Streptococcus pneumoniae* 670-6B | GCA_000147095.1 |
|  | Streptococcus pneumoniae G54, | GCA_000019825.1 |
|  | Streptococcus pneumoniae P1031 | GCA_000019005.1 |
|  | Streptococcus pneumoniae D39 | GCA_000014365.2 |
|  | Streptococcus pneumoniae 70585 | GCA_000018965.1 |
|  | Streptococcus pneumoniae JJA | GCA_000018985.1 |
|  | *Streptococcus pneumoniae* INV104 | GCA_000210975.1 |
|  | Streptococcus pneumoniae OXC141 | GCA_000210975.1 |
|  | [Streptococcus pneumoniae INV200](https://www.ncbi.nlm.nih.gov/nuccore/FQ312029.1/) | GCA_000210935.1 |
| ***Enterococcus faecium*** | | |
|  | Enterococcus faecium DO | GCA_000174395.2 |
|  | Enterococcus faecium Aus0085 | GCA_000444405.1 |
|  | *Enterococcus faecium* Aus0004 | GCA_000250945.1 |
|  | *Enterococcus faecium* SRR24 | GCA_009734005.2 |
|  | *Enterococcus faecium VRE* | GCA_009697285.1 |
|  | *Enterococcus faecium UCH1* | GCA_023204955.1 |
|  | *Enterococcus faecium E1* | GCA_001886635.1 |
|  | *Enterococcus faecium*  NY11066 | GCA_022699565.1 |
|  | *Enterococcus faecium* JHP80 | GCA_026073415.1 |
| ***Streptococcus gallolyticus*** | | |
|  | *Streptococcus gallolyticus* [FDAARGOS_755](https://www.ncbi.nlm.nih.gov/genome/2742?genome_assembly_id=906560) | [GCA_013267695.1](https://www.ncbi.nlm.nih.gov/assembly/GCA_013267695.1) |
|  | *Streptococcus gallolyticus* [XH2168](https://www.ncbi.nlm.nih.gov/genome/2742?genome_assembly_id=2121514) | [GCA_027474865.2](https://www.ncbi.nlm.nih.gov/assembly/GCA_027474865.2) |
|  | *Streptococcus gallolyticus* [ICDDRB-NRC-S1](https://www.ncbi.nlm.nih.gov/genome/2742?genome_assembly_id=260392) | [GCA_001477575.1](https://www.ncbi.nlm.nih.gov/assembly/GCA_001477575.1) |
|  | *Streptococcus gallolyticus* [UCN34](https://www.ncbi.nlm.nih.gov/genome/2742?genome_assembly_id=172621) | [GCA_000027185.1](https://www.ncbi.nlm.nih.gov/assembly/GCA_000027185.1) |
|  | *Streptococcus gallolyticus* [ATCC 43143](https://www.ncbi.nlm.nih.gov/genome/2742?genome_assembly_id=172623) | [GCA_000270145.1](https://www.ncbi.nlm.nih.gov/assembly/GCA_000270145.1) |
|  | *Streptococcus gallolyticus* [ATCC BAA-2069](https://www.ncbi.nlm.nih.gov/genome/2742?genome_assembly_id=172622) | [GCA_000203195.1](https://www.ncbi.nlm.nih.gov/assembly/GCA_000203195.1) |
|  | *Streptococcus gallolyticus* [TX20005](https://www.ncbi.nlm.nih.gov/genome/2742?genome_assembly_id=1648582) | [GCA_019021805.1](https://www.ncbi.nlm.nih.gov/assembly/GCA_019021805.1) |
